# Emotional and behavioural problems and non-suicidal self-injury in preadolescent children: complementary insights from child and parent reports

**DOI:** 10.3389/fpsyt.2026.1973448

**Published:** 2026-10-20

**Authors:** Tengfen Yang, Minghui Li, Hang Yu, Xiaofei Hou, Guoli Yan, Boxuan Li, He Ma, Ling Sun, Huifang Yin, Guangming Xu

**Affiliations:** Tianjin Anding Hospital, Mental Health Center of Tianjin Medical University, Tianjin, China

**Keywords:** child and parent reports, China, emotional and behavioural problems, non-suicidal self-injury, preadolescent children

## Abstract

**Objective:**

Given the scarcity of research on preadolescent non-suicidal self-injury (NSSI) and its clinical significance, this study examined its prevalence and associations with emotional/behavioural problems, and the differences and complementarity between child and parent reports in a Chinese community sample.

**Methods:**

In total, 1,923 children (mean age = 11.01 years, SD = 0.79; 49.8% male) were recruited via convenience sampling from eight primary schools in Tianjin. NSSI was assessed by child self-report. Emotional and behavioural problems were measured using child and parent versions of the Strengths and Difficulties Questionnaire (SDQ). Multivariable logistic regression, formal Wald tests, hierarchical regression, and ROC curve analyses were used to examine associations, compare informant-specific effects, assess incremental validity, and evaluate model performance.

**Results:**

Of the participants, 484 (25.2%) reported at least one NSSI episode in the past six months. Logistic regression revealed overlapping and discrepant patterns: emotional symptoms and hyperactivity were significant in both reports; peer interaction problems were unique to child reports, while conduct problems were unique to parent reports. However, formal comparisons using joint models and Wald tests showed that only emotional symptoms, hyperactivity, and peer interaction problems showed significantly larger effects in child reports (all *p* < 0.001). No significant differences were found for conduct problems or prosocial behaviour. Child reports provided greater incremental information beyond parent reports (Δ*R*² = 0.124 vs. 0.014, both *p* ≤ 0.001). The child-report model showed significantly better discrimination than the parent-report model (AUC = 0.753 vs. 0.659; DeLong test: *Z* = -7.75, *p* < 0.001).

**Conclusion:**

NSSI was common among preadolescents in this sample. The overlapping yet discrepant patterns suggest that child and parent reports provide complementary information: children appear more attuned to internalising symptoms and peer distress, whereas parents appear more sensitive to conduct problems. These findings highlight the value of multi-informant assessment in school-based screening.

## 1 Introduction

Non-suicidal self-injury (NSSI) is defined as the deliberate and direct destruction of body tissue without explicit suicidal intent. It has been included in the Diagnostic and Statistical Manual of Mental Disorders, Fifth Edition (DSM-5) as a condition warranting further research and is associated with severe functional impairment, including decreased academic performance and increased susceptibility to psychiatric disorders (1). Although NSSI is most prevalent during adolescence and young adulthood (2), a substantial proportion of individuals who self-injure report an onset before adolescence. Specifically, an estimated 5.1% – 24% of individuals with a history of NSSI began the behaviour between 11 and 13 years of age (3, 4). Early-onset NSSI is of particular clinical concern. It is associated with a more severe clinical presentation, increased risk of recurrent self-injury and hospitalisation, and greater treatment difficulty once the behaviour has become established (5, 6). However, research specifically targeting children aged 9–12 years remains limited. This developmental period is widely recognised as preadolescence.

The prevalence and distribution of NSSI are known to vary by age and gender, with higher rates consistently observed among females and a peak during mid-adolescence (7, 8). This developmental pattern may explain why the majority of existing research has focused on adolescents and young adults. Younger populations, particularly children aged 9–12 years, remain substantially understudied (9, 10). Importantly, emotional and behavioural problems have been consistently identified as key correlates of NSSI across developmental stages. NSSI has been conceptualised as a maladaptive strategy to regulate negative emotional states and alleviate psychological distress (11, 12). This regulatory framework may be particularly relevant during preadolescence. This period is characterised by a maturational imbalance between amygdala-driven emotional reactivity and still-immature prefrontal cognitive control systems (13). Empirical evidence supports this framework, showing that internalising difficulties (e.g., depression, anxiety) and externalising problems (e.g., aggression, conduct problems) are both prospectively associated with increased NSSI risk (14–16). Although the association between emotional/behavioural problems and NSSI is well-established in adolescents, it remains unclear whether similar patterns hold among children aged 9–12 years, and whether these associations differ depending on who provides the information about the child’s emotional and behavioural functioning.

Previous studies on this topic have several limitations. First, the majority of NSSI research has been conducted in secondary school settings, focusing primarily on adolescents and young adults. Younger populations, particularly children aged 9–12 years, remain relatively understudied. Second, most existing studies have relied on a single informant to assess emotional and behavioural problems, typically either the child’s self-report or the parent-report alone. This is a notable gap because parent-child agreement on emotional and behavioural difficulties is known to be low, particularly for internalising problems such as emotional symptoms, which parents may not readily observe (17, 18). To obtain a more comprehensive and accurate assessment of children’s mental health, it is essential to incorporate information from both children and parents. Moreover, most existing evidence originates from Western samples. Emotional expression and help-seeking are culturally shaped, especially in East Asian contexts where academic pressure is intense. Therefore, cross-cultural validation is critically needed (19). To address these gaps, the present study aimed to: (1) estimate the prevalence of NSSI in children aged 9–12 years; (2) examine the associations of child-reported and parent-reported emotional and behavioural problems with NSSI; and (3) compare the patterns of association across informants and examine whether child and parent reports provide complementary information in relation to NSSI.

## 2 Materials and methods

### 2.1 Participants and procedure

A school-based cross-sectional survey was conducted in December 2021 in Dongli District, Tianjin, China. Participants were recruited from eight primary schools using a convenience sampling method. Students in grades 4 to 6 were eligible to participate. Inclusion criteria were: (1) current enrollment in a participating primary school; (2) ability to understand and complete the questionnaire independently; and (3) provision of child assent and written parental consent. Exclusion criteria were: (1) significant reading impairments or uncooperative behaviour; (2) diagnosed severe psychiatric disorders or organic brain conditions; and (3) incomplete or invalid responses.

Prior to the formal survey, we contacted the principals and headteachers of the eight participating schools to obtain institutional approval and administrative support. Headteachers from each class received standardised training on the survey procedures to ensure consistency in data collection across schools. A dual-channel recruitment strategy was adopted to reach parents and students. Parents were initially informed of the study’s objectives and procedures by class teachers via WeChat. Following this, students took home paper-based informed consent forms for their parents to sign. Only students who provided their own assent and returned signed parental consent forms were enrolled in the study. Data were collected using paper questionnaires, with separate versions for students and parents. Students completed the child-report questionnaires in their classrooms during school hours, with trained teachers and research assistants present to ensure privacy and prevent discussion. Parents completed the parent-report questionnaires at home and were asked to seal the completed forms in the provided envelopes, which were then returned to the school by their children. After data collection, each participating family received a summary health feedback report. Students who were identified as having potential mental health concerns or self-injurious behaviours were referred to the school counselling service for further evaluation and support. The study protocol was approved by the Tianjin Mental Health Center Ethics Committee (approval number: 2021-42). All procedures were conducted in accordance with the ethical standards of the Declaration of Helsinki and its later amendments. Written informed consent was obtained from the parents or legal guardians of all participants, and child assent was also secured prior to data collection.

Parent-child dyads were matched using unique identification numbers. Each child received a unique identification number prior to data collection, and the same number was printed on both the child and parent questionnaires, allowing paired data to be linked after collection. Questionnaires that could not be matched (i.e., missing either the child or parent version) or that lacked the identification number were excluded.

All questionnaire data were independently entered by two researchers using SPSS 25.0, and any discrepancies between the two entries were resolved through verification against the original paper forms. A total of 4,206 students were initially enrolled in the study. Dyads were excluded if: (1) parent- or child-report SDQ data were missing (n = 2,181); (2) DSHI-s data were missing (n = 6); or (3) questionnaires had more than 2 missing items on the DSHI-s or more than 2 missing items on any SDQ subscale, or showed uniform response patterns across all items (n = 96). The final analytic sample comprised 1,923 parent-child dyads (**Supplementary Table S2**). For retained questionnaires, missing values were minimal and were replaced at the item level using the mode of the corresponding item. Mode substitution was chosen because the SDQ and DSHI-s items are categorical/ordinal in nature. A sensitivity analysis using complete-case analysis yielded consistent results (**Supplementary Table S1**).

### 2.2 Instruments

#### 2.2.1 Deliberate self-harm inventory-short

NSSI was assessed using the short form of the Deliberate Self-Harm Inventory (DSHI-s). The original DSHI was developed by Gratz (20) for use in adult populations and was later revised and simplified by Lundh et al. (21) for use in adolescent populations. The DSHI-s is a self-report questionnaire comprising 16 items that assess the occurrence and frequency of a range of intentional self-injurious behaviours. Each item is rated on a frequency scale from 1 (“never”) to 4 (“three or more times”). In the present study, children who endorsed at least one intentional self-injurious behaviour during the preceding six months were operationally classified as having self-reported NSSI. Suicidal intent was not assessed separately; therefore, the present assessment cannot definitively establish that every endorsed episode occurred in the absence of suicidal intent. Accordingly, NSSI in this study should be understood as an operational classification based on self-reported self-injurious behaviours, rather than as a clinically or diagnostically confirmed condition. This operational approach is consistent with previous studies in elementary school children, in which NSSI was classified based on self-reported self-injurious behaviours using simplified self-harm measures (22). Previous studies using modified versions of the DSHI in Chinese children and adolescents have reported good internal consistency (Cronbach’s alpha = 0.90–0.99) (23).

#### 2.2.2 The strengths and difficulties questionnaire

Emotional and behavioural problems were assessed using both the child self-report and parent-report versions of the Strengths and Difficulties Questionnaire (SDQ). The SDQ was developed by Robert Goodman as a brief behavioural screening tool for children and adolescents (24). The Chinese version was translated and adapted by Du et al., and has demonstrated good reliability and validity in Chinese samples (25–27). The SDQ consists of 25 items divided into five subscales: emotional symptoms, conduct problems, hyperactivity/inattention, peer interaction problems, and prosocial behaviour. Each item is rated on a 3-point Likert scale (0 = not true, 1 = somewhat true, 2 = certainly true), with five items (7, 11, 14, 21, and 25) reverse-scored. Each subscale comprises five items, yielding a total score ranging from 0 to 10 for each domain. Higher scores on the first four subscales indicate greater difficulties, whereas a higher score on the prosocial subscale reflects stronger social competence. Although the self-report SDQ is recommended for children aged 11 years and above, previous studies have demonstrated acceptable psychometric properties in children as young as 8–10 years when administered in school settings (28, 29). In the present study, trained research assistants were available during questionnaire administration to provide standardized clarification of item wording when children requested assistance.

### 2.3 Statistical analysis

Normality of continuous variables was assessed using the Kolmogorov-Smirnov test. As the SDQ subscale scores were not normally distributed, they are presented as medians and interquartile ranges (IQRs). Categorical variables are presented as frequencies and percentages. Between-group comparisons (NSSI vs. non-NSSI) were performed using Mann-Whitney U tests for continuous variables and chi-square tests for categorical variables using SPSS version 25.0.

All five SDQ subscales were entered simultaneously into the multivariable logistic regression models based on prior theoretical and empirical evidence. Child-reported and parent-reported SDQ scores were analysed in separate models, each with NSSI status as the dependent variable, and both models were adjusted for age and gender (grade was not included due to its high conceptual overlap with age in this developmental period). The results are reported as odds ratios (*OR*) with 95% confidence intervals (*CI*).

To facilitate comparison of effect sizes across informants, all SDQ subscale scores were standardized (z-scores) prior to logistic regression. Standardized ORs therefore represent the change in NSSI odds per 1-SD increase in each subscale. To formally test whether the effects of corresponding subscales differed between parent and child reports, joint logistic regression models were fitted including both parent- and child-reported scores for each subscale simultaneously, adjusting for age and gender. The equality of coefficients was tested using Wald tests. Given the paired nature of the data (parent and child reports from the same dyad), the covariance between coefficients within the joint model captures within-dyad correlation. Multiple comparisons were controlled using the Benjamini-Hochberg false discovery rate (FDR). Multicollinearity among predictors was examined using variance inflation factors (VIF), and all VIF values were below 2 (maximum = 1.80), indicating no problematic multicollinearity.

Incremental validity was assessed using hierarchical logistic regression. Two sequences were tested. In the first sequence, covariates (age and gender) were entered in Step 1, child-reported SDQ in Step 2, and parent-reported SDQ in Step 3. In the second sequence, parent-reported SDQ was entered in Step 2 and child-reported SDQ in Step 3. Model comparisons were conducted using likelihood ratio tests (Omnibus Tests of Model Coefficients) and changes in Nagelkerke *R*² (Δ*R*²).

To compare the overall discriminative performance of the child-report and parent-report SDQ models, we calculated the area under the receiver operating characteristic curve (AUC) for each model following logistic regression using R software (version 4.5.3) with the pROC package. Differences between the two AUCs were tested using DeLong’s method for correlated ROC curves (30). Tied values in predicted probabilities were observed due to the discrete nature of the predictors; however, given the large sample size, the bias in AUC estimation is expected to be negligible, and the DeLong test remains valid for correlated ROC curves in this context. A two-tailed *p*-value of < 0.05 was considered statistically significant.

A sensitivity analysis using complete-case analysis was conducted to examine the robustness of the main findings, and the results were consistent with the primary analyses. All statistical analyses were performed using SPSS version 25.0 and R software version 4.5.3. The pROC and car packages in R were used for AUC comparison and Wald tests, respectively.

## 3 Results

A total of 1,923 parent-child dyads were included in the final analysis. Of these, 958 (49.8%) were male, and the mean age was 11.01 years (SD = 0.79). Of the 1,923 participants, 484 (25.2%) reported at least one NSSI episode during the past six months and were classified as the NSSI group; the remaining 1,439 (74.8%) were classified as the non-NSSI group. Among the 484 children in the NSSI group, the most commonly reported form was carving words, pictures, designs, or other marks into the skin (n = 317, 65.5%). This was followed by cutting the wrist, arms, or other areas of the body (n = 164, 33.9%) and severely scratching oneself to the extent that scarring or bleeding occurred (n = 147, 30.4%). Other frequently reported forms included biting oneself to the extent that the skin was broken (n = 105, 21.7%), preventing wounds from healing (n = 73, 15.1%), and sticking sharp objects such as needles, pins, or staples into the skin (n = 70, 14.5%). Less common forms included burning with a cigarette, lighter, or match (n = 21, 4.3%), and self-harm leading to hospitalisation or medical treatment (n = 15, 3.1%). A full breakdown of all 16 DSHI-s items is provided in **Supplementary Table S3**.

The NSSI group had a higher proportion of females compared with the non-NSSI group; however, this difference was not statistically significant (χ² = 3.626, *p* = 0.057). The mean age of the NSSI group was 10.89 years (SD = 0.778), which was slightly lower than that of the non-NSSI group (11.01 years, SD = 0.808; *t* = 2.751, *p* = 0.006). As shown in **Table 1**, both child-reported and parent-reported SDQ scores were significantly higher in the NSSI group than in the non-NSSI group across all four problem subscales (emotional symptoms, conduct problems, hyperactivity, and peer interaction problems; all *p* < 0.01), whereas prosocial behaviour scores were significantly lower in the NSSI group (*p* < 0.001).

**Table 1:** Characteristics of participants by NSSI status.

Characteristic	Subcategory	No-NSSI (*n* = 1,439)	NSSI (*n* = 484)	*Z/*χ²/*t*	*P*
gender	Male	735 (51.1)	223 (46.1)	3.63	0.057
	Female	704 (48.9)	261 (53.9)		
Age		11.01 ± 0.81	10.89 ± 0.78	2.75	0.006^*^
Self-reported	Emotional symptoms	1 (0–2)	2 (1–4)	-14.21	<0.001^**^
	Conduct problems	1 (0–2)	2 (1–3)	-9.96	<0.001^**^
	Hyperactivity	2 (0–3)	3 (2–4)	-11.98	<0.001^**^
	Peer interaction problems	2 (1–3)	3 (2–4)	-8.49	<0.001^**^
	Prosocial behaviour	9 (7–10)	8 (6–10)	-7.17	<0.001^**^
Parent-reported	Emotional symptoms	1(0–2)	1.5 (0–3)	-7.47	<0.001^**^
	Conduct problems	1 (0–2)	1 (1–2)	-7.34	<0.001^**^
	Hyperactivity	3 (1–4)	4 (2–5)	-8.20	<0.001^**^
	Peer interaction problems	2 (1–3)	2 (1–4)	-3.11	0.002^*^
	Prosocial behaviour	9 (7–10)	8 (6–10)	-5.41	<0.001^**^

Continuous variables are presented as mean ± SD or median (IQR), depending on data distribution. Group comparisons were performed using independent-samples t-tests (for age), Mann-Whitney U tests (for SDQ subscale scores), or chi-square tests (for gender). NSSI, non-suicidal self-injury; SDQ, Strengths and Difficulties Questionnaire; IQR, interquartile range. ^*^p<0.05; ^**^p<0.001.

To examine the associations between SDQ subscales and NSSI, two separate multivariable logistic regression models were constructed adjusting for age and gender. Because all SDQ subscale scores were standardized, the ORs represent the change in NSSI odds per 1-SD increase in each subscale (**Table 2**). In the parent-report model, emotional symptoms (*OR* = 1.26, 95% CI: 1.13-1.42, *p* < 0.001), conduct problems (*OR* = 1.18, 95% CI: 1.05-1.33, *p* = 0.006), and hyperactivity (*OR* = 1.25, 95% CI: 1.10-1.42, *p* = 0.001) were significantly associated with NSSI. Peer interaction problems (*OR* = 0.99, 95% CI: 0.89-1.12, *p* = 0.923) and prosocial behaviour (*OR* = 0.90, 95% CI: 0.80-1.01, *p* = 0.082) were not significant. Parent-reported peer interaction problems differed significantly between the NSSI and non-NSSI groups in univariable comparisons (**Table 1**). However, this association became non-significant in the multivariable model, likely because its variance was shared with other SDQ subscales entered simultaneously. In the child-report model, emotional symptoms (*OR* = 1.66, 95% CI: 1.47-1.88, *p* < 0.001), hyperactivity (*OR* = 1.39, 95% CI: 1.22-1.59, *p* < 0.001), and peer interaction problems (*OR* = 1.24, 95% CI: 1.10-1.40, *p* < 0.001) were significantly associated with NSSI, whereas conduct problems (*OR* = 1.07, 95% CI: 0.94-1.22, *p* = 0.310) and prosocial behaviour (*OR* = 0.94, 95% CI: 0.83-1.06, *p* = 0.279) were not.

**Table 2:** Standardized odds ratios of parent- and child-reported SDQ subscales for NSSI (separate models).

SDQ subscale	Parent-reported *OR* (95%CI)	*P*	Child-reported *OR* (95% CI)	*P*
Emotional symptoms	1.26 (1.13–1.42)	<0.001^**^	1.66 (1.47–1.88)	<0.001^**^
Conduct problems	1.18 (1.05–1.33)	0.006^*^	1.07 (0.94–1.22)	0.310
Hyperactivity	1.25 (1.10–1.42)	0.001^*^	1.39 (1.22–1.59)	<0.001^**^
Peer interaction problems	0.99 (0.89–1.12)	0.923	1.24 (1.10–1.40)	<0.001^**^
Prosocial behaviour	0.90 (0.80–1.01)	0.082	0.94 (0.83–1.06)	0.279

NSSI, non-suicidal self-injury; SDQ, Strengths and Difficulties Questionnaire; OR, odds ratio; CI, confidence interval. All models were adjusted for age and gender. SDQ subscale scores were standardized; ORs represent the change in NSSI odds per 1-SD increase in each subscale. As these models are cross-sectional, they cannot establish temporal or causal relationships. ^*^p<0.05; ^**^p<0.001.

To formally test whether the effects of corresponding subscales differed between informants, joint models were fitted including both parent- and child-reported scores. Wald tests were used to compare the equality of coefficients (**Table 3**). The effect of child-reported emotional symptoms was significantly larger than that of parent-reported emotional symptoms (χ² = 30.17, *p* < 0.001, FDR-adjusted *p* < 0.001). Similarly, significant differences were observed for hyperactivity (χ² = 12.32, *p* < 0.001, FDR-adjusted *p* < 0.001) and peer interaction problems (χ² = 19.12, *p* < 0.001, FDR-adjusted *p* < 0.001). No significant differences were found for conduct problems (χ² = 2.42, *p* = 0.119, FDR-adjusted *p* = 0.149) or prosocial behaviour (χ² = 1.04, *p* = 0.306, FDR-adjusted *p* = 0.306). Incremental validity was assessed using hierarchical logistic regression (**Table 4**). In the first sequence, when child-reported SDQ was entered first, adding parent-reported SDQ significantly improved model fit (*ΔR²* = 0.014, χ² = 21.24, *p* = 0.001). In the second sequence, when parent-reported SDQ was entered first, adding child-reported SDQ led to a substantially larger improvement in model fit (*ΔR²* = 0.124, χ² = 179.49, *p* < 0.001). The final combined model explained 21.0% of the variance in NSSI (Nagelkerke *R²* = 0.210).

**Table 3:** Formal comparisons of parent- vs. child-reported SDQ subscale effects (joint models with Wald tests).

SDQ subscale	Parent-reported *OR* (95% CI)	Child-reported *OR* (95% CI)	Wald χ²	FDR-adjusted *P*
Emotional symptoms	1.16 (1.04–1.30)	1.95 (1.74–2.19)	30.17	<0.001^**^
Conduct problems	1.33 (1.19–1.47)	1.51 (1.36–1.68)	2.42	0.149
Hyperactivity	1.23 (1.09–1.39)	1.75 (1.56–1.97)	12.32	<0.001^**^
Peer interaction problems	1.05 (0.94–1.17)	1.57 (1.41–1.76)	19.12	<0.001^**^
Prosocial behaviour	0.85 (0.76–0.94)	0.77 (0.69–0.86)	1.04	0.306

OR, odds ratio; CI, confidence interval; SDQ, Strengths and Difficulties Questionnaire; NSSI, non-suicidal self-injury; FDR, Benjamini-Hochberg false discovery rate. Each joint model included both parent- and child-reported scores for a given subscale simultaneously, adjusting for age and gender. All SDQ subscale scores were standardized; ORs represent the change in NSSI odds per 1-SD increase in each subscale. Wald tests compared the equality of coefficients between informants. ^*^p<0.05; ^**^p<0.001.

**Table 4:** Incremental validity of child- and parent-reported SDQ in predicting NSSI (hierarchical logistic regression).

Step	Predictors	-2LL	Nagelkerke *R*²	Δ*R²*	χ²	*p*
Sequence 1: child first						
1	Covariates	2159.18	0.008	–	10.67	0.005^*^
2	+ Child SDQ	1896.57	0.196	0.188	262.61	<0.001^**^
3	+ Parent SDQ	1875.33	0.210	0.014	21.24	0.001^*^
Sequence 2: parent first						
1	Covariates	2159.18	0.008	–	10.67	0.005^*^
2	+ Parent SDQ	2054.82	0.086	0.078	104.36	<0.001^**^
3	+ Child SDQ	1875.33	0.210	0.124	179.49	<0.001^**^

SDQ, Strengths and Difficulties Questionnaire; −2LL, −2 log-likelihood; ΔR², change in Nagelkerke R² from the previous step. Covariates, age and gender. χ² values are from likelihood ratio tests (Omnibus Tests of Model Coefficients). Sequence 1 shows the incremental validity of parent-reported SDQ after child-reported SDQ; Sequence 2 shows the incremental validity of child-reported SDQ after parent-reported SDQ. ^*^p<0.05; ^**^p<0.001.

We further compared the overall discriminative performance of the two informant-based models using ROC curve analysis (**Figure 1**). The child-report SDQ model showed moderate discrimination (AUC = 0.753, 95% CI: 0.732-0.779), whereas the parent-report SDQ model showed acceptable discrimination (AUC = 0.659, 95% CI: 0.638-0.694). A DeLong test for correlated ROC curves confirmed that this difference was statistically significant (ΔAUC = 0.094, *Z* = -7.75, *p* < 0.001; **Table 5**).

**Figure 1:**
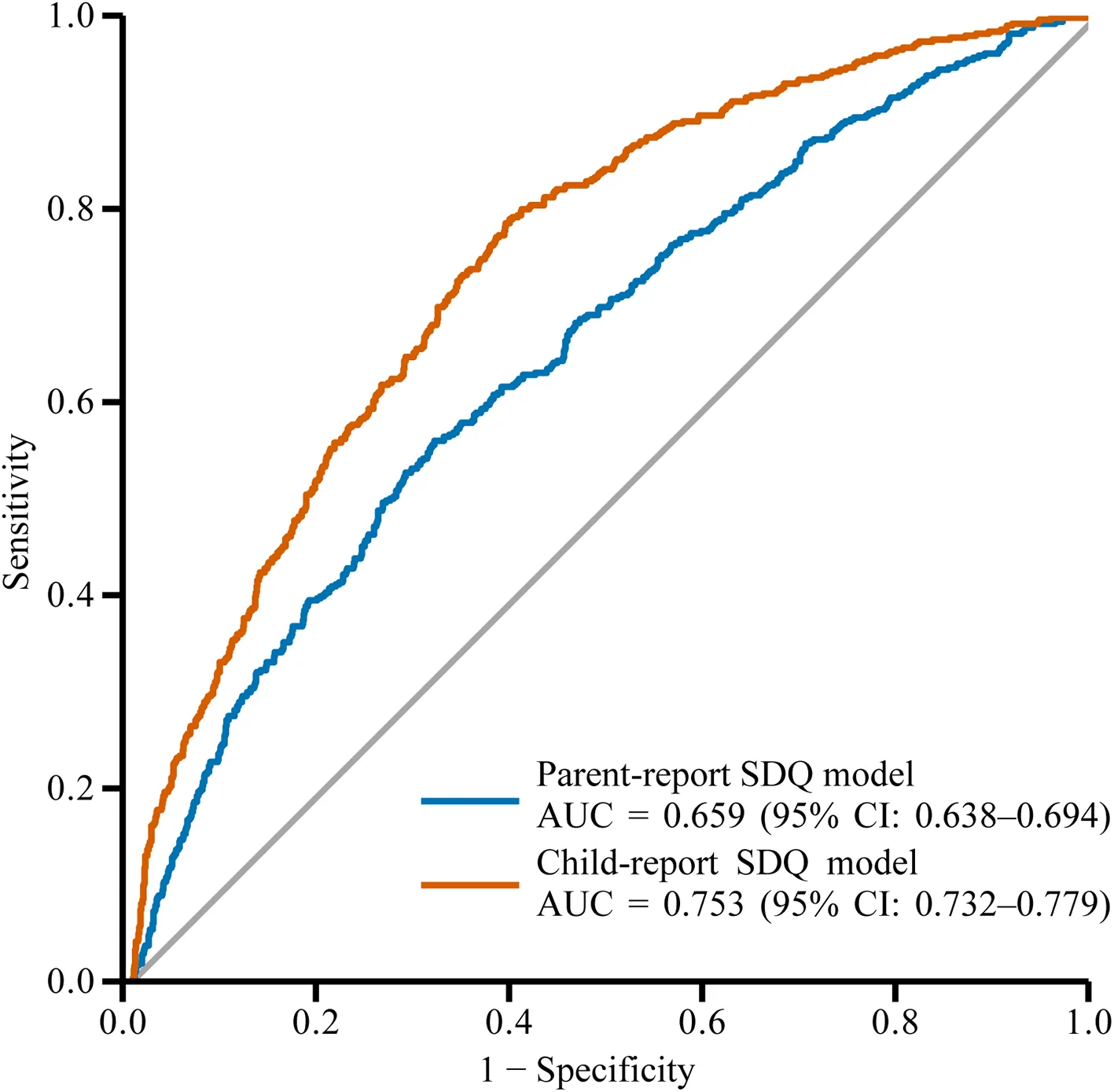
ROC curves for child-report and parent-report SDQ models.

**Table 5:** Discriminative performance of parent- and child-reported SDQ models.

Model	AUC (95% CI)	ΔAUC	DeLong *Z*	DeLong *p*
Parent-report SDQ	0.659 (0.638–0.694)	Ref	–	–
Child-report SDQ	0.753 (0.732–0.779)	0.094	-7.75	<0.001^**^

AUC, area under the receiver operating characteristic curve; CI, confidence interval; SDQ = Strengths and Difficulties Questionnaire. ΔAUC = child-report AUC − parent-report AUC. Ref = reference model. DeLong’s test for correlated ROC curves was used to compare the two AUCs. ^**^p<0.001.

## 4 Discussion

To date, population-based data on NSSI among children aged 9–12 years remain sparse, particularly in non-Western contexts. This study examined the prevalence of NSSI and its association with emotional and behavioural problems in a community sample of Chinese preadolescents, using both child self-reports and parent reports. Our findings reveal that NSSI is not uncommon in this developmental window, with a six-month prevalence of 25.2%, and is significantly associated with both internalising and externalising difficulties. Notably, the distinct patterns of association across informants provide insights into NSSI in this age group.

Our prevalence estimate (25.2%) is broadly consistent with prior Chinese school-based studies using the same instrument (29.26%) (31). However, it is markedly higher than rates reported in Western community samples of similar age bands (7–9%) (15, 32). This discrepancy may reflect differences in measurement instruments, sample sources, cultural context, and survey timing, rather than a single explanatory factor. Our regression analyses revealed that both internalising problems (i.e., emotional symptoms) and externalising problems (i.e., conduct problems and hyperactivity) were associated with increased odds of NSSI in our sample of children aged 9–12 years. This is consistent with a substantial body of literature demonstrating that emotional and behavioural difficulties are significant correlates of NSSI across developmental periods (10, 33). Several possible explanations may account for these associations. Emotionally distressed individuals may engage in NSSI as a means of regulating negative affect. The physical pain associated with self-injury may serve to distract from emotional distress (34). Regarding externalising problems, hyperactivity and impulsivity have been prospectively associated with elevated NSSI risk (35). Similarly, conduct problems have been prospectively associated with NSSI (36). In addition, peer interaction problems were significantly associated with NSSI in our self-report data and have been identified as a correlate of self-injurious behaviour in previous studies (36, 37).

Our formal comparisons using joint models and Wald tests showed that child-reported effects were significantly larger than parent-reported effects for emotional symptoms, hyperactivity, and peer interaction problems, whereas no significant differences were found for conduct problems or prosocial behaviour. Parent-reported emotional symptoms (*OR* = 1.26) and peer interaction problems (*OR* = 0.99, non-significant) showed weaker associations with NSSI compared with child-reported emotional symptoms (*OR* = 1.66) and peer interaction problems (*OR* = 1.24). This pattern suggests that parents may under-recognise the internal distress associated with preadolescent NSSI and perceive mainly observable behaviours, such as conduct problems and hyperactivity (parent-report *OR* = 1.18 and 1.25). In contrast, child-reported peer interaction problems and emotional symptoms may capture experiences that occur mainly in school and peer settings and may be less readily observed by parents (38, 39). ROC curve analyses further supported this informant discrepancy. The child-report model yielded a significantly higher AUC than the parent-report model (AUC = 0.753 vs. 0.659; DeLong test: *Z* = -7.75, *p* < 0.001). This indicates that children’s self-perceived emotional and social difficulties were more strongly associated with NSSI status than parents’ observations. However, the AUC of 0.753 indicates moderate rather than high discriminative ability, and the child-report model should not be over-interpreted as a highly accurate screening tool. Further validation is needed before it can be applied for screening purposes. These findings should be interpreted within the cross-sectional design and cannot establish causality or identify the underlying mechanisms of NSSI.

Our findings have several practical implications for the screening and prevention of NSSI in children aged 9–12 years. First, the high prevalence observed in this community sample underscores the need for routine mental health screening in primary school settings, rather than limiting assessments to clinical populations. Second, the distinct patterns of association revealed by child self-reports and parent reports highlight the complementary value of a multi-informant approach. Relying solely on parent reports may miss critical risk indicators, particularly peer relationship difficulties and emotional distress, which children’s own self-reports capture best. Conversely, child self-reports may underestimate externalising problems such as conduct difficulties, which parents are better positioned to observe.

## 5 Conclusions

In this community sample of children aged 9–12 years, emotional and behavioural difficulties reported by both children and parents were associated with concurrent self-reported NSSI. Formal between-informant comparisons indicated stronger child-reported associations for emotional symptoms, hyperactivity, and peer interaction problems, whereas no significant between-informant differences were observed for conduct problems or prosocial behaviour. Child and parent reports also provided partially non-redundant information, although the incremental contribution of parent reports beyond child reports was modest. Because suicidal intent was not directly assessed, NSSI was operationally classified on the basis of self-reported self-injurious behaviours and should not be interpreted as clinically confirmed NSSI. The cross-sectional design, substantial loss of incomplete parent-child dyads, shared reporting source for child SDQ and NSSI, and lack of independent model validation limit causal, generalisability, and screening interpretations.

## 6 Strengths and limitations

Several strengths of this study should be noted. First, although a few previous studies have examined NSSI in Chinese school-aged samples, our study focuses specifically on children aged 9–12 years, providing new prevalence data and correlational evidence for this developmental period. Second, unlike most prior research that relied on a single informant, we employed both child self-reports and parent reports (SDQ) to assess emotional and behavioural problems, allowing for a more comprehensive evaluation of child mental health. Several limitations should also be acknowledged. First, the cross-sectional design precludes any inferences about causality or temporal directionality; the associations we observed between emotional/behavioural problems and NSSI should be interpreted as correlational, and future longitudinal studies are needed to clarify the causal pathways. Second, the sample was drawn from a single province in China (Tianjin) using convenience sampling, which may limit the generalisability of our findings to other regions or populations. In particular, the prevalence estimate of 25.2% reflects self-reported NSSI in this specific sample and should not be directly generalised to all Chinese children. Third, NSSI was assessed exclusively by child self-report using the DSHI-s and was not independently verified through clinical interview or another informant. In addition, suicidal intent was not directly assessed. Therefore, although endorsed behaviours were operationally classified as NSSI, we cannot confirm that every reported episode was strictly non-suicidal. The prevalence reported here should consequently be interpreted as the prevalence of self-reported NSSI as operationalised by the DSHI-s rather than clinically confirmed NSSI. This limitation also restricts direct comparison with studies that explicitly assessed and excluded suicidal intent. Future studies should incorporate developmentally appropriate assessment of suicidal intent and clinically validated NSSI measures in children. Fourth, the age appropriateness of the measures should be considered. The self-report SDQ is officially recommended for children aged 11 years and above, and the DSHI-s was originally developed for adolescents. Although previous studies have demonstrated acceptable psychometric properties in children as young as 8–10 years, psychometric validation specifically in Chinese children aged 9–12 years remains limited for both instruments. Future studies should validate these measures in this age group. Fifth, the present study did not assess the specific functions of NSSI (e.g., emotion regulation, social signalling), which may have distinct risk factor profiles and should be explored in future research. Sixth, our regression models adjusted only for age and gender. Other potential confounders—such as family socioeconomic status, parental mental health, academic stress, peer victimisation, and parenting styles—were not included due to the scope of the survey. Therefore, the observed odds ratios may be subject to residual confounding and should be interpreted with caution. Although our findings are robust to the covariates we examined, future studies with more comprehensive assessments are warranted to determine whether the observed associations remain stable after controlling for a broader range of clinical and demographic variables. In addition, because both NSSI and the child-reported SDQ were assessed through child self-report, whereas the parent SDQ was obtained from a different informant, shared method variance may have contributed to the stronger associations and higher apparent discrimination observed for the child-report model. Therefore, the present findings should not be interpreted as demonstrating the inherent superiority or greater accuracy of child reports.

## Data Availability

The data that support the findings of this study are available from the corresponding author upon reasonable request. Requests to access the datasets should be directed to Guangming Xu (xugm@tmu.edu.cn).
