# Supplementary material for: Emotional and behavioural problems and non-suicidal self-injury in preadolescent children: complementary insights from child and parent reports
Source: Front Psychiatry. 2026 Oct 20;17:1973448. doi: 10.3389/fpsyt.2026.1973448 (PMC13729380; doi:10.3389/fpsyt.2026.1973448)
Supplement: Supplementary file 1 [file Table1.docx]

Supplementary Material

## Supplementary Tables

**Supplementary Table S1.** Sensitivity analysis using complete-case analysis for standardized ORs, Wald tests, and incremental validity.

Panel A. Standardized odds ratios (separate models).

|  | Parent-reported *OR* (95%CI) | *P* | | Child-reported *OR* (95% CI) | | *P* | |
| --- | --- | --- | --- | --- | --- | --- | --- |
| Emotional symptoms | 1.27 (1.14–1.43) | | <0.001^**^ | | 1.69 (1.50–1.91) | | <0.001^**^ |
| Conduct problems | 1.18 (1.04–1.33) | | 0.008^*^ | | 1.06 (0.94–1.21) | | 0.343 |
| Hyperactivity | 1.26 (1.11–1.43) | | <0.001^**^ | | 1.39 (1.22–1.58) | | <0.001^**^ |
| Peer interaction problems | 1.00 (0.89–1.12) | | 0.995 | | 1.24 (1.10–1.39) | | 0.001^*^ |
| Prosocial behaviour | 0.90 (0.80–1.02) | | 0.089 | | 0.94 (0.83–1.05) | | 0.247 |

Panel B. Formal comparisons (joint models with Wald tests).

|  | Wald *χ*² | *P* | FDR-adjusted *P* |
| --- | --- | --- | --- |
| Emotional symptoms | 30.17 | <0.001^**^ | <0.001^**^ |
| Conduct problems | 2.42 | 0.126 | 0.157 |
| Hyperactivity | 12.32 | 0.001^*^ | 0.001^*^ |
| Peer interaction problems | 19.12 | <0.001^**^ | <0.001^**^ |
| Prosocial behaviour | 1.04 | 0.306 | 0.306 |

Panel C. Incremental validity (hierarchical logistic regression).

| Step | Predictors | -2LL | Nagelkerke *R*² | Δ*R*² | *χ²* | *p* |  |
| --- | --- | --- | --- | --- | --- | --- | --- |
| **Sequence 1: Child first** |  |  |  |  |  |  | |
| 1 | Covariates | 2165.57 | 0.008 | – | 10.79 | 0.005^*^ | |
| 2 | + Child SDQ | 1897.71 | 0.199 | 0.191 | 267.86 | <0.001^**^ | |
| 3 | + Parent SDQ | 1875.72 | 0.214 | 0.015 | 22.00 | 0.001^*^ | |
| **Sequence 2: Parent first** |  |  |  |  |  |  | |
| 1 | Covariates | 2165.57 | 0.008 | – | 10.79 | 0.005^*^ | |
| 2 | + Parent SDQ | 2057.92 | 0.088 | 0.080 | 107.65 | <0.001^**^ | |
| 3 | + Child SDQ | 1875.72 | 0.214 | 0.126 | 182.20 | <0.001^**^ | |

***Note.*** *All analyses were based on complete-case analysis (n = 1,923). SDQ = Strengths and Difficulties Questionnaire; NSSI = non-suicidal self-injury; OR = odds ratio; CI = confidence interval; −2LL = −2 log-likelihood; ΔR² = change in Nagelkerke R² from the previous step; FDR = false discovery rate. Covariates = age and gender. In Panel A, all SDQ subscale scores were standardized; ORs represent the change in NSSI odds per 1-SD increase in each subscale. In Panel B, each joint model included both parent- and child-reported scores for a given subscale simultaneously; Wald tests compared the equality of coefficients between informants; FDR-adjusted p values refer to the Wald test p values after Benjamini-Hochberg correction for multiple comparisons. In Panel C, χ² values are from likelihood ratio tests (Omnibus Tests of Model Coefficients); Sequence 1 shows the incremental validity of parent-reported SDQ after child-reported SDQ, and Sequence 2 shows the incremental validity of child-reported SDQ after parent-reported SDQ. The pattern of significant findings was consistent with the main analysis.* ^*^*p<0.05;* ^**^*p<0.001.*

**Supplementary Table S2.** Demographic characteristics of included and excluded participants.

|  | Included (n = 1,923) | Excluded (n = 2,283) | *t* /*χ²* | *P* |
| --- | --- | --- | --- | --- |
| Age, mean (SD) | 10.98 (0.81) | 9.47 (0.89) | 55.98 | <0.001^**^ |
| Gender, n (%) |  |  | 3.58 | 0.058 |
| Male | 936 (49.4) | 1,109 (52.4) |  |  |
| Female | 959 (50.6) | 1,008 (47.6) |  |  |
| Grade, n (%) |  |  | 2761.80 | <0.001^**^ |
| Grade 4 | 129 (6.7) | 1,905 (88.9) |  |  |
| Grade 5 | 958 (49.9) | 63 (2.9) |  |  |
| Grade 6 | 834 (43.4) | 175 (8.2) |  |  |

***Note.*** *Age was compared using independent-samples t-test; gender and grade were compared using chi-square tests. Analyses were restricted to participants with available data on the respective variables (age: n = 1,919 included, n = 2,127 excluded; gender: n = 1,895 included, n = 2,117 excluded; grade: n = 1,921 included, n = 2,143 excluded). Included = dyads retained in the final analysis; Excluded = dyads excluded due to missing SDQ, missing DSH-s, or invalid responses.*

**Supplementary Table S3.** Frequency of different NSSI methods among children who reported at least one NSSI episode (n = 484).

| NSSI method | *n* (%) |
| --- | --- |
| 1. Intentionally cut wrist, arms, or other areas of the body | 164 (33.9) |
| 2. Intentionally burned self with a cigarette, lighter, or match | 21 (4.3) |
| 3. Intentionally carved words, pictures, designs, or other marks into the skin | 317 (65.5) |
| 4. Intentionally severely scratched self, to the extent that scarring or bleeding occurred | 147 (30.4) |
| 5. Intentionally bit self, to the extent that the skin was broken | 105 (21.7) |
| 6. Intentionally rubbed sandpaper on the body | 43 (8.9) |
| 7. Intentionally dripped acid onto the skin | 5 (1.0) |
| 8. Intentionally used bleach, comet, or oven cleaner to scrub the skin | 10 (2.1) |
| 9. Intentionally stuck sharp objects (e.g., needles, pins, staples) into the skin | 70 (14.5) |
| 10. Intentionally rubbed glass into the skin | 27 (5.6) |
| 11. Intentionally broke own bones | 11 (2.3) |
| 12. Intentionally banged head against something, causing a bruise to appear | 33 (6.8) |
| 13. Intentionally punched self, causing a bruise to appear | 40 (8.3) |
| 14. Intentionally prevented wounds from healing | 73 (15.1) |
| 15. Did anything else to hurt self that was not asked about in the questionnaire | 92 (19.0) |
| 16. Intentionally hurt self in any of the above ways, leading to hospitalisation or injury requiring medical treatment | 15 (3.1) |

***Note.*** *Denominators are the 484 children who reported at least one NSSI episode in the past six months. Participants could report multiple methods, so percentages do not sum to 100%. NSSI = non-suicidal self-injury.*
